# Complete Genome Sequence of a Multidrug-Resistant *Acinetobacter baumannii* Isolate Obtained from a Mexican Hospital (Sequence Type 422)

**DOI:** 10.1128/genomeA.00583-16

**Published:** 2016-06-23

**Authors:** Semiramis Castro-Jaimes, Abraham David Salgado-Camargo, Lucía Graña-Miraglia, Luis Lozano, Paola Bocanegra-Ibarias, Patricia Volkow-Fernández, Jesus Silva-Sanchez, Santiago Castillo-Ramírez, Miguel A. Cevallos

**Affiliations:** a Centro de Ciencias Genómicas, Programa de Genómica Evolutiva, Universidad Nacional Autónoma de México, Cuernavaca, Morelos, Mexico; b Departmento de Enfermedades Infecciosas, Laboratorio de Microbiología, Instituto Nacional de Cancerología, Secretaría de Salud (SSA), Cuernavaca, Morelos, Mexico; c Departamento de Diagnóstico Epidemiológico, Centro de Investigaciones sobre Enfermedades Infecciosas, Instituto Nacional de Salud Pública, Cuernavaca, Morelos, Mexico; d Hospital Universitario de Nuevo León, Monterrey, Nuevo León, Mexico

## Abstract

*Acinetobacter baumannii* has emerged as a dangerous nosocomial pathogen, particularly for severely ill patients in intensive care units and patients with hematologic malignancies. Here, we present the complete genome sequence of a multidrug-resistant *A. baumannii* isolate, recovered from a Mexican hospital and classified as sequence type 422 according to the multilocus sequence typing Pasteur scheme.

## GENOME ANNOUNCEMENT

*Acinetobacter baumannii* is a Gram-negative bacterium that has emerged as a dangerous pathogen worldwide, causing a variety of nosocomial infections, especially in severely ill patients in intensive care units and patients with hematologic malignancies on chemotherapy (1). *A. baumannii* isolates are resistant to most of the currently used antibiotics, including carbapenems, and they are able to survive for extended periods of time under a broad range of environmental conditions, such as dry surfaces. These characteristics promote *A. baumannii* outbreaks in the hospital setting. Most of the isolates causing outbreaks worldwide belong to the international clonal lineages I and II (2), clonal complexes 1 and 2, respectively, as defined by the multilocus sequence typing (MLST) Pasteur scheme (3).

Here, we present the complete genome sequence and annotation of *A. baumannii* 3207, an isolate not related to the international clonal lineages but instead classified as sequence type 422 according to the MLST Pasteur scheme (3). This multidrug-resistant isolate was recovered from bronchoalveolar lavage fluid of a 45-year-old female patient admitted to the Hospital Universitario de Nuevo León, Mexico, a tertiary care center, in August 2008.

The complete genome of *A. baumannii* 3207 was determined on a PacBio RSII platform. To do this, a large insert library (20 to 25 kb) was constructed and sequenced, using one SMRT cell with a P6 polymerase and C4 chemistry combination (P6-C4) with a 180-min movie. The SMRT cells produced 551,632,314 post-filter polymerase reads with a mean read length of 10,718 bp. Subreads were assembled *de novo* using the PacBio RS hierarchical genome assembly process (HGAP) protocol version 3 in SMRT analysis version 2.3 (Pacific Biosciences) (4). To improve regions of low coverage, a library with a 2 × 300-bp paired-end configuration was sequenced on an Illumina MiSeq platform. The sequencing yielded 3,117,310 reads, resulting in 909,042,928 bp. A hybrid assembly was constructed with the Illumina MiSeq reads and PacBio RII subreads using SPAdes version 3.5.0 (5). The contigs corresponding to the chromosome and the plasmids were circularized using a Perl script available at https://github.com/jfass/apc. Functional annotation was performed with the NCBI Prokaryotic Genome Annotation Pipeline.

The *A. baumannii* 3207 genome consists of a circular chromosome (3,998,013 bp) and two plasmids: pAba3207a (13,479 bp) and pAba3207b (80,547 bp). The chromosome has six rRNA operons, 74 tRNAs, and 3,674 coding sequences (CDSs) and embraces most of the antibiotic resistance genes, conferring resistance to aminoglycosides, beta-lactams, carbapenems (blaOXA-65), cephalosporins, fosmidomycin, fusaric acid, phenicols, and tetracyclines. The smallest plasmid has 19 CDSs, including one carbapenem resistance gene (blaOXA-58), whereas pAba3207b has 97 CDSs.

This and a previous report (6) are the first steps toward understanding the genomic diversity of *A. baumannii* in Mexico, not only at some loci but at the genome level. However, to get a more comprehensive picture, comparative analyses of many more isolates are warranted.

### Nucleotide sequence accession numbers.

The genome sequence of isolate 3207 was deposited in GenBank under the accession numbers CP015364, CP015365, and CP015366.
